# Neurostimulation as a Method of Treatment and a Preventive Measure in Canine Drug-Resistant Epilepsy: Current State and Future Prospects

**DOI:** 10.3389/fvets.2022.889561

**Published:** 2022-06-16

**Authors:** Marta Nowakowska, Muammer Üçal, Marios Charalambous, Sofie F. M. Bhatti, Timothy Denison, Sebastian Meller, Gregory A. Worrell, Heidrun Potschka, Holger A. Volk

**Affiliations:** ^1^Research Unit of Experimental Neurotraumatology, Department of Neurosurgery, Medical University of Graz, Graz, Austria; ^2^Department of Small Animal Medicine and Surgery, University of Veterinary Medicine Hannover, Hanover, Germany; ^3^Small Animal Department, Faculty of Veterinary Medicine, Small Animal Teaching Hospital, Ghent University, Merelbeke, Belgium; ^4^Department of Engineering Science, Institute of Biomedical Engineering, University of Oxford, Oxford, United Kingdom; ^5^Department of Neurology, Mayo Clinic, Rochester, MN, United States; ^6^Faculty of Veterinary Medicine, Institute of Pharmacology, Toxicology and Pharmacy, Ludwig-Maximilians-University, Munich, Germany

**Keywords:** drug-resistant epilepsy, dogs, vagus nerve stimulation, deep brain stimulation, transcranial magnetic stimulation, seizure, epileptogenesis

## Abstract

Modulation of neuronal activity for seizure control using various methods of neurostimulation is a rapidly developing field in epileptology, especially in treatment of refractory epilepsy. Promising results in human clinical practice, such as diminished seizure burden, reduced incidence of sudden unexplained death in epilepsy, and improved quality of life has brought neurostimulation into the focus of veterinary medicine as a therapeutic option. This article provides a comprehensive review of available neurostimulation methods for seizure management in drug-resistant epilepsy in canine patients. Recent progress in non-invasive modalities, such as repetitive transcranial magnetic stimulation and transcutaneous vagus nerve stimulation is highlighted. We further discuss potential future advances and their plausible application as means for preventing epileptogenesis in dogs.

## Introduction

Epilepsy is the most common neurological brain disorder affecting both humans and non-human animals, with a prevalence in the human population of 0.64% in the active form or 0.76% with cases in remission (lifetime prevalence) (1), and in dogs 0.6–0.75% of the general dog population (2). However, the mere presence of genetically very homogenous purebred populations favors a more frequent occurrence of epilepsy in some canine breeds. Here, the prevalence can range from 3% up to 18% (2) or even 33% as described in a family of Belgian shepherd dogs (3). The high prevalence rates underscore the relevance of this condition for veterinary practice.

Epilepsy poses a significant challenge for veterinary and human medicine, in part because of the high rates of resistance to first and second line anti-seizure medications. The occurrence of drug-resistant epilepsy (DRE) has been reported in 13.7% of the community out-patient and 36.3% of the clinic-based human population (4), and similar numbers are assumed to apply in dogs (5). Many hypotheses exist regarding the pathophysiology of DRE, including alterations in blood brain barrier's multidrug transporter expression, pharmacokinetics, pharmacodynamics, genetic variability, functional changes of neural networks and intrinsic severity of the disease, as well as involvement of inflammatory processes (6, 7). Most third line therapeutic approaches aim to circumvent some of those challenges. Common treatment approaches in human medicine include dietary approaches, brain surgery and neurostimulation (6, 8). While the first two approaches are relatively easy to implement in veterinary practice (9, 10), surgery and neurostimulation remain problematic because of their cost, time, and high level of skills required. However, the growing body of evidence for efficacy of neurostimulation techniques in human patients is raising awareness and interest in this therapeutic approach among veterinary practitioners. Therefore, it is of interest to know, which techniques have already been applied in canine patients with DRE, to understand their advantages and disadvantages, and to develop a road-map for their further development and assessment in canine patients.

First mentions of neurostimulation as a therapeutic method date back to the first century CE. At that time, electric fish attachment to skin was used to relief pain in patients (11). Advances in understanding of physics of electricity in the late nineteenth and early twentieth century revived interest in neurostimulation, which became a popular topic in the 1950s and 1960s when various devices, including those for epilepsy management, were developed (12–17). However, although significant technological improvements have been made in recent decades, our understanding of the mechanism of action of neurostimulation in the context of many diseases remains vague.

Neurostimulation can be performed both in the peripheral and in the central nervous system. While first one is e.g., performed in cases of neuropathic pain (18, 19), for nerve regeneration after injury (20) and to re-establish sensation in people with prostheses (21), central stimulation serves alleviation of symptoms of e.g., tremor diseases (22–24), neuropsychiatric disorders (25–27), pain (28, 29) and epilepsy (30–32). Vagus nerve stimulation (VNS), deep brain stimulation (DBS), and transcranial magnetic stimulation (TMS) are the current methods applied and described in veterinary medicine. Therefore, the review article has focused on these three therapy options.

Neurostimulation exerts effects on nervous tissue at cellular, molecular and structural levels. Mathematical modeling of high frequency stimulation in neural networks revealed its stabilizing influence on cells (33). Neural circuits showed reduced susceptibility to sudden transitions into oscillations usually marking the onset of a seizure. Moreover, inhibitory cells were recruited more strongly than excitatory cells, putting the system in an “anti-seizure state” (33). This mechanism may be the basis for acute seizure termination after application of high frequency stimulation. Brain stimulation also led to changes in connectivity of the brain inside and outside of epileptic foci and different protocols led to promotion or suppression of circuit synchronicity (34, 35).

Stimulation of neural tissue alters not only its electrical properties but also its chemical microenvironment. Several studies describe its modulatory influence on release and production of neurotransmitters, extracellular vesicles, brain-derived neurotrophic factor (BDNF) and on receptor function (36–40). Similarly, neurostimulation promotes glial cell activation, astrocytic signaling and proliferation of neuronal progenitor cells (41, 42). This might serve as a double-edged sword in the process of epileptogenesis, starting regenerative processes in the brain on one hand, which on the other hand might lead to creation of hyperexcitable networks, when they turn abnormal, as observed in rodent models of epilepsy and epileptogenesis (43, 44). However, early concerns about therapeutic electrical brain stimulation kindling human brain has not been seen in the class-I evidence trials of responsive neural stimulation (RNS) (45) and deep brain stimulation of anterior nucleus of thalamus (DBS) (46) in long-term human trials.

As a matter of course, the main goal of electrical stimulation of the epileptic brain is better seizure control. As can be seen from the neuronal network studies, this can be achieved either by stopping a developing seizure or by preventing its occurrence in the first place. The goal can be achieved either by targeted stimulation ideally before a seizure manifests (using sophisticated prediction algorithms), or by providing a cumulative long-term anti-seizure effect of regular continuous stimulations. Since long-term complete freedom from seizures is rarely achieved with electrical stimulation, therapeutic success can be difficult to define and quantify. Moreover, it often depends on patient's age, sex, and individual variability (47). Particularly important in human epilepsy is the impact of epilepsy on mood, memory, and quality of life. While rarely achieving complete seizure freedom the class-I evidence trials in humans demonstrate improved quality of life.

The need for individualized decisions is also evident when it comes to selection of the optimal method of neurostimulation: not every patient will be eligible for surgery or anesthesia, so electrode implantations might be contraindicated in these cases. Understanding the advantages and disadvantages of the most commonly used neurostimulation methods will certainly be beneficial to many veterinary neurologists. Learning from veterinary researchers conducting pilot studies in canine patients and from experienced human neurologists applying neurostimulation approaches in their clinics will be useful for applying neurostimulation in their veterinary research and practice.

## Vagus Nerve Stimulation

Vagus nerve stimulation (VNS) as treatment of human epilepsy was first introduced in 1988 (48); however, initial trials of external stimulation of the vagus nerve date more than 100 years earlier (49). Even before the first implantation in humans, Zabara managed to attenuate seizures evoked by injections of strychnine or pentylenetetrazole (PTZ) in dogs (50), which paved the way for further clinical trials. VNS got approval for management of epilepsy in Europe in 1994 and in the USA in 1997 (49) and currently, it is being used by more than 100,000 patients worldwide (51).

The exact mechanism of action of VNS in epilepsy has not been fully elucidated yet, but anatomy and physiology of the vagus nerve gives insight into possible processes involved. Vagus nerve, the longest cranial nerve, arises in the nucleus ambiguous of the medulla, exits the cranium *via* the jugular foramen and extends into the neck, thorax and abdomen, where it supplies muscles and inner organs. Over 80% of vagal fibers carry sensory information from the viscera toward the brain (afferent fibers), while only around 20% of fibers are responsible for motor signaling (efferent fibers) (49, 52). Afferents terminate in the nucleus of solitary tract, which projects to multiple brain regions, among which the most crucial for the anti-seizure effect seem to be locus coeruleus and raphe nuclei (52). These regions are strongly activated by VNS and they are heavily engaged in production of neurotransmitters, such as noradrenaline and serotonin, which further stimulate interneurons to release gamma-amino butyric acid (GABA), increasing seizure threshold of neurons. Other potential mechanisms of anti-seizure action of VNS include changes in blood flow in regions correlating with seizure reduction (52–54), up-regulation of neurotrophin production (55) and anti-inflammatory effects (56, 57).

Histologically, the vagus nerve is composed of A-, B-, and C-fibers, the first two types being myelinated. Myelination and diameter of fibers (the largest in A-fibers, the smallest in C-fibers) directly translates in their various stimulation thresholds. Recordings from de-sheathed vagus nerves in healthy dogs placed amplitude thresholds to evoke action potentials at 0.4 mA for A-fibers, for fast B-fibers: 1.6 mA, for slow B-fibers: 3.8 mA and for C-fibers: 17 mA (58). Since current amplitude used for invasive VNS in dogs ranges in literature from 0.25 to 1.5 mA (Table 1), it can be assumed the effects of stimulation are mostly associated with activation of A- and fast B-fibers, consisting of motor and sensory afferent fibers (58).

**Table 1:** Current neurostimulation parameters and outcomes in veterinary medicine.

**Authors (year)**	**Intervention**	**Study design**	**Participants**	**Inclusion criteria**	**Parameters**	**Main outcomes**	**Side effects**
Muñana et al. (59)	VNS	Double-blinded placebo-controlled crossover study	10 owner-kept dogs, randomized allocation	Onset 1–5 years, at least 1 year seizure history, frequency at least 5 seizures/months, no longer seizure-free than 2 weeks or clusters 1/month; current treatment with ASD (normal serum conc.), at least 15 kg	0.25 to 1.0 mA, 30 Hz, pulse width 500 μs, ON: 30 s, OFF: 5 min	13 week treatment—no difference; last 4 days—decrease in seizure frequency (34.4%)	Intraoperative: bradycardia, asystole, apnea; postoperative: seroma, device migration, Horner's syndrome
Martlé et al. (60)	VNS	Placebo-controlled crossover study, single-blinded for PTZ test	8 experimental Beagle dogs, randomized paradigms	No history of neurological or other diseases	Output current: as high as possible without cough; ON: 7 s, OFF: 18 s; *rapid cycling standard VNS*: 30 Hz, pulse width 500 μs; *microburst VNS*: 300 Hz, pulse width 500 μs, 3 pulses/burst, inter-burst interval: 0.4 s	Increase of CSF norepinephrine conc. 1 h after stim. in standard (67%) and microburst (76%); no difference in dopamine and serotonin conc.; no difference in PTZ threshold	Muscle tremors and spasm of left thoracic limb (one dog)
Martlé et al. (53)	VNS	Single-blinded placebo-controlled crossover study	10 experimental Beagle dogs, randomized paradigms	No history of neurological or other diseases	Output current: as high as possible without cough; ON: 7 s, OFF: 18 s; *rapid cycling standard VNS*: 30 Hz, pulse width 500 μs; *microburst VNS*: 300 Hz, pulse width 500 μs, 3 pulses/burst, inter-burst interval: 0.4 s	Hypoperfusion of left frontal and right parietal cortices in microburst	Seroma, hoarseness, Horner's syndrome (exclusion criteria)
Harcourt-Brown and Carter (61)	VNS	Non-blinded prospective cohort study	16 owner-kept dogs, non-randomized allocation	Tier II diagnosis of idiopathic epilepsy	0.25 to 1.5 mA (*slow ramping*: increase every 1–3 weeks; *fast ramping*: 8–12 h), 30 Hz, pulse width 250 μs, ON: 7 s (30 s), OFF: 1.8 min (5 min)	14 dogs reached 1.5 mA (72 days fast vs. 77 days slow)—no effectiveness of seizure frequency decrease was evaluated	Seroma, coughing, muscle fasciculation, abnormal tongue position and dysphagia (one dog), lead twisting and breaking
Hirashima et al. (62)	VNS	Case report	1 owner-kept Shetland sheepdog	Tier III diagnosis of idiopathic epilepsy	0.25 to 0.75 mA, 20 Hz, pulse width 250 μs, ON: 30 s, OFF: 5 min (1.8 min)	87% reduction of focal to generalized tonic-clonic seizures; 89% reduction of focal to generalized tonic-clonic seizures clusters; 76% reduction of days with a focal to generalized tonic-clonic seizures; no generalization of focal seizures upon magnet use	Cough during stim.
Robinson et al. (63)	Non-invasive VNS	Non-blinded prospective cohort study	14 owner-kept dogs, randomized allocation	Tier I or tier II diagnosis of idiopathic epilepsy	60 mA (at the skin level), 5 5,000 Hz pulses repeated at 25 Hz for 90–120 s 3 times a day	Four dogs with seizure frequency reduction ≥50%, 9/14 reduction, 1/14 no change, 4/14 increase	Hoarseness and trembling of left thoracic limb (one dog), progressive behavioral changes (one dog)
Zamora et al. (64)	DBS	Case report	1 owner-kept mixed-breed dog	Tier II diagnosis of idiopathic epilepsy	*Basal stim. during awakefulness and active*	Prevention of SE and reduction of coherent cluster	Involuntary motion during HF stimulation
					*phases*: 13 Hz 0.5 (day) or 0.7 (night) mA; *elevated stim. during sleep phases:* 13 Hz, 1.3 mA; *high-amplitude HF stim. to terminate seizures*: burst of 130 Hz, 1.5 mA	seizures during the follow-up phase of 7 months	
Charalambous et al. (65)	rTMS	*Trial I*: single-blinded placebo-controlled prospective study; *trial II*: open-labeled uncontrolled prospective study	*Trial I*: 12 owner-kept dogs, randomized allocation; *trial II*: 5 owner kept-dogs, non-randomized (dogs from trial I sham group)	Tier I or tier II diagnosis of idiopathic epilepsy	1 Hz, 90 pulses, 18 trains/day, 5 days	*Trial I*: reduction in the monthly frequency of seizures and seizure day; *trial II*: as above, effect lasting 4 months	No treatment-related side effects were reported

*ASM, anti-seizure medication; conc., concentration; CSF, cerebrospinal fluid; DBS, deep brain stimulation; HF, high-frequency; PTZ, pentylenetetrazole; rTMS, repeated transcranial magnetic stimulation; SE, status epilepticus; stim., stimulation; VNS, vagus nerve stimulation*.

In human neurology, VNS is indicated, as a third line treatment of epilepsy, when candidates meet following criteria: medically refractory seizures; adequate trials of at least 2 anti-seizure drugs; exclusion of non-epileptic events; and ineligibility for epileptogenic focus resection surgery (66). Usually, it is applied in cases of intractable focal and secondarily generalized tonic-clonic epilepsy, in epilepsy of generalized onset (including atonic seizures) and in epileptic syndromes (54). The implantable device consists of a helical electrode placed around the cervical part of the vagus nerve, a connective lead and a pulse generator, usually localized in a subclavicular region (57). Usually in epilepsy treatment, VNS is applied to the left vagus nerve due to its innervation of the atrioventricular node of the heart. The right vagus nerve innervates the sinoatrial node, the stimulation of which could lead to severe cardiac adverse effects (67). Additionally, care is taken to place the VNS electrodes distal to the superior and inferior cervical cardiac branches of the vagus nerve.

**Figure 1:**
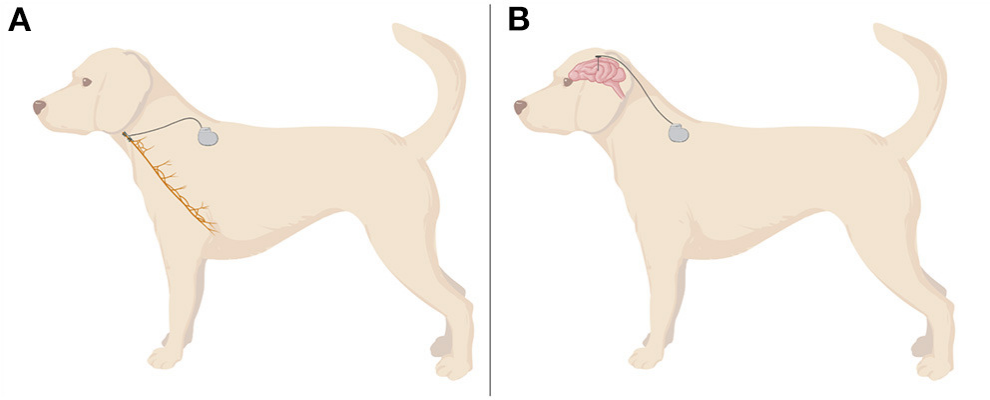
A demonstrative illustration of assembly of invasive VNS **(A)** and DBS **(B)** in a dog. VNS electrodes are mostly wrapped around the cervical portion of left vagus nerve, whereas DBS electrodes are usually placed in thalamic nuclei. Wires and a controlling device are usually located in a dorsal cervical region. Created with BioRender.com.

In dogs, it is impossible to spare the cardiac branches from stimulation, because they leave the nerve more distally in the thoracic cavity. Therefore, the electrodes are wrapped around the left vagosympathetic trunk, as both nerves are fused in the cervical region in this species (68) (Figure 1). Consequently, additional sympathetic stimulation and influence on the heart cannot be excluded. During the surgery, the cathode is placed rostrally, the anode in the middle and anchor tether on the caudal portion (68). This configuration (proximal cathode/distal anode) stimulates predominantly afferent vagal fibers, while proximal anode/distal cathode leads mostly to the stimulation of efferents (69). Simultaneously, it does not influence vagal fibers' threshold to evoke action potentials, which only remain sensitive to the amplitude of current used for stimulation (58, 69). Pulse generator can be located dorsally on the left cervical region (68) or on thorax (61), underneath muscular fascia or muscle. Subcutaneous placing is discouraged to avoid migration and seroma formation at the surgery site (68).

In people, the vagus nerve is mostly stimulated in an open-loop fashion: duty cycle (ON and OFF periods) with additional extra stimulation delivered by an external magnet swipe delivered by the patient or caregiver for acute seizures (57). Available closed-loop stimulators utilize sophisticated algorithms to detect seizure events based on ictal cardiac activity associated with seizures (70, 71). Comparison between open- and closed-loop approaches in one cohort study of pediatric patients suggests a better response to VNS after 2 years of treatment, especially among children with generalized epilepsy (71). In dogs, closed-loop VNS has not been studied yet, the evidence from humans suggests however, it could prove beneficial, especially in long term. Additionally, it could decrease the burden of caretakers and veterinary staff, since they would not have to apply additional stimulation with external magnet swipe at the seizure onset.

Human patients with epilepsy undergoing VNS experience a decrease of seizure intensity, seizure duration and a shortening of the post-ictal period (54, 57, 72, 73). The main outcome crucial for the success of anti-seizure therapy, namely reduction of seizure frequency by ≥50%, is reported in ~60% of patients (73) and this effect increases with time (49), often requiring more than half a year for maximal effect (74). Long-term studies demonstrated an improvement of seizure frequency reduction after 1 year of treatment as compared to 3 months stimulation (75, 76) and it reached its peak after 2 years of VNS (77). Secondary effects associated with VNS include improvement of mood, cognition and memory (52, 78–80) as well as lowering of anxiety (78, 81). More recently VNS has been shown to reduce the incidence of sudden unexplained death in epilepsy (SUDEP) (82). Evidence of VNS effects in canine epilepsy is much less abundant than of those gathered from human patients, nevertheless this mode of stimulation has already proved beneficial for dogs with DRE. In the first clinical study published in 2002 by Muñana et al. 10 dogs with DRE demonstrated a decrease in mean seizure frequency by 34.4% in the last 4 weeks of 13-week long therapy (59). Four of nine dogs showed a reduction of seizure frequency by ≥50% (so-called good responders) in this period, while two of them responded in that way during the whole study period (59). This study has shown VNS to reduce seizure frequency in a subpopulation of dogs with DRE, but it is unknown if the seizure suppressing effect increases further, like in people, in the first 6 to 8 months or if VNS remains effective long term. Hirashima et al. recently published a case study with a longer follow-up period (62). A 5-year old Shetland sheepdog had focal seizures and generalized seizures with focal onset for 4 years before implantation of the VNS system. The study followed the patient from 3 months before the implantation up to 1 year after the beginning of the stimulation and described in detail protocol adjustments and their outcomes. After a 1-year follow-up the authors noticed 87% reduction in generalized seizures with focal onset, 89% reduction of focal-to-generalized cluster seizures and 76% decrease of days in which focal-to-generalized seizures appeared (62). Moreover, focal seizures did not progress into generalization, when the owner activated the VNS system externally with a magnet at their onset (62). The cognitive effect of VNS has not been described in canine epileptic patients to date. However, the treatment improved their overall quality of life (62), even in cases when the seizure frequency was not reduced (59).

The most common adverse effects of invasive VNS in humans include postoperative infection (3–6% of cases), vocal cord paresis and lower facial nerve palsy (54, 57, 74). Cardiac side effects such as bradycardia or asystole usually happen during the intraoperative device testing and cease after protocol modification (54). In dogs, side effects associated with VNS include seroma at the site of implantation (53, 59, 68), coughing (62) and muscle twitching during the treatment (60). Cardiac adverse effects such as bradycardia, asystole, and apnea were observed only during intraoperative device testing (59). A prospective cohort study by Harcourt-Brown et al. examined in detail short-term adverse effect in dogs suffering from DRE, reporting cough as the most common one, having developed in 11 out of 14 dogs (61). To eliminate severe coughs (mild and moderate coughing few to several times a day was considered tolerable) the authors introduced protocol modifications based on guidelines published for humans (83). Briefly, when intolerable (harsh or accompanied by retching) coughing was encountered, the authors first changed duty cycle (ON-time: 30 s to 7 s; OFF-time; 5 min to 1.8 min), in the case of no effect they reduced frequency (25 to 20 Hz), and as the last step they reduced current to the highest tolerable level (61). A similar approach was used by Hirashima et al. and proved beneficial for the examined patient (62). Recently, a prospective, double-blind clinical trial aiming to develop new titration protocols has been conducted in human DRE-patients (84). It could lead to better optimization of stimulation parameters and perhaps offer better adjustment strategy for veterinary patients as well.

In recent years, popularity in human epileptology was gained by transcutaneous non-invasive VNS (nVNS), which can be applied either on skin of pinna (auricular branch of the vagus) or along the nerve trajectory on the neck (57). Transcutaneous approaches require higher current intensity, while other stimulation parameters (pulse width, frequency and duty cycles) remain usually similar to invasive VNS (85). Although extensive clinical evidence regarding nVNS is still lacking, data from preliminary human trials showed that this method engages the same neural pathways as invasive VNS (86) and yields seizure reduction in patients with DRE (87–89). nVNS requires less frequent stimulation schedules, which leads to overall less adverse effects (88). Most frequently reported adverse effects of nVNS are headache, ear/facial pain and skin irritation at the stimulation site (57). nVNS constitutes an attractive alternative approach for veterinary medicine, especially for patients not eligible for surgery. In a study published in 2020 by Robinson et al., 14 dog patients with refractory idiopathic epilepsy underwent 8- or 16-week long VNS treatment with a non-invasive stimulator along the cervical portion of the left vagus nerve (63). Nine dogs showed reduction in seizure frequency compared to baseline, among which four were considered good responders (reduction of seizure frequency by ≥50%) (63). Authors also mention that one patient did not show any change and four experienced an increase in seizure frequency (63). More studies would be welcome to elucidate long-term applicability and safety of nVNS in canine epilepsy. Additionally, auricular stimulation could prove beneficial, especially in patients who do not accept manipulations around their neck. However, diverse anatomy of canine ears could negatively influence standardization of such study.

VNS application extends beyond neurological diseases: a growing body of clinical evidence from human patients indicates its suitability for treatment of chronic heart failure (90, 91) or inflammatory diseases such as Crohn's disease (92, 93) or rheumatoid arthritis (94, 95). Recently, nVNS has been proposed and applied to patients with respiratory symptoms of COVID-19 to modulate their inflammatory response (96–98). VNS improved cardiovascular parameters and decreased plasma and heart tissue biomarkers associated with heart failure in a canine model of heart failure (99) and lead to weight loss in dogs (100) and minipigs (101). VNS is undoubtedly a powerful tool, which, if understood and applied properly, could bring a new value to human and veterinary medicine and lead to bidirectional translation of methodology and applications.

## Deep Brain Stimulation

Intracranial deep brain stimulation (DBS) in human patients with epilepsy has been investigated for many targets including: cerebellum, subthalamic nucleus, centromedium thalamus and hippocampus (102). DBS is an approved therapy for human focal epilepsy in Europe, USA, Canada, South America and Australia targeting the anterior nuclei of the thalamus (ANT). Responsive neurostimulation (RNS) of the epileptogenic focus and network is approved in the USA (45, 103). The latter approach is further discussed below detailing the closed-loop approach interfering with ongoing ictal activity.

One major question addressed in experimental studies and clinical pilot studies related to the choice of the optimal anatomical target (104). Several potential target regions have been assessed in experimental and clinical pilot studies. Among these the ANT has been selected for a large double-blind randomized multicenter trial. In this initial trial (the SANTE trial) a gradual increase in efficacy was observed in the group of patients with a high frequency 145 Hz bilateral stimulation (46). In this group, the reduction in seizure frequency at 3 month amounted to 40.4% as compared to 14.5% in the control group without stimulation. However, group differences did not reach significance, when considering the entire 3-month stimulation phase. Trial data resulted in approval of ANT for treatment of drug-resistant epilepsy in patients with focal-onset seizures in Europe, Australia and South America, but was delayed in the US until 2018. Long-term follow-up studies provided evidence that efficacy may further increase with prolonged stimulation (105).

Adverse effects described in the initial clinical trial and subsequent studies comprised surgery-related risks including infection, hemorrhage and pain, and stimulation-related effects including headache, sleep disturbance, increased anxiety, and depression (16).

Despite the growing amount of human clinical data and the increasing interest in ANT deep brain stimulation for management of DRE, there a still various open questions concerning the mechanisms, patient selection, electrode placement techniques, and optimal programming (106, 107). In line with the role of the ANT as a network hub in limbic circuits, evidence exists that patients with temporal lobe epilepsy show a favorable response as compared to patients with frontal lobe epilepsy and epilepsies with other locations. Further clinical factors in patient selection include patient preference, operability, history of psychogenic seizure and of psychiatric disorders (106). According to an expert consensus contraindications for ANT deep brain stimulation comprise progressive etiology, psychiatric disorders, MRI contraindications (e.g., older generations electric implants such as cardiac pacemakers, insulin pumps as well as metal foreign bodies), and incomplete seizure diaries (106).

Considering the impact of high frequency stimulation on ictogenesis different mechanisms are discussed. These comprise preferential activation of inhibitory GABAergic neurons, alterations in extracellular potassium concentrations, desynchronization of neuronal activities, and reduction of the recruitment of neurons to epileptic rhythmic activity (108). Recently, attempts with continuous stimulation paradigms (109–111) and multiple thalamic targets using 4-lead devices (112) have been undertaken.

Recently a first case study has been published reporting deep brain stimulation in a canine patient with a progressive increase in seizure severity with frequent cluster seizures and repeated escalation of seizure activity into status epilepticus (64). Considering evidence that the centromedian nucleus of the thalamus (CMNT) can play a role during the early or late phase of an epileptic seizure the stimulation electrode was placed in this thalamic nucleus (113). Case reports in human patients with super-refractory status epilepticus have already suggested DBS of the CMNT or ANT as a rescue therapy for super-refractory status epilepticus (114–119).

Building on this clinical experience, Zamora et al. (64) have applied a multi-scale, rhythm entrained stimulation of the CMNT in a 4-year old, mixed breed dog suffering from idiopathic drug-refractory epilepsy with seizure occurrence associated with awake/sleep phases (Figure 1). The individualized approach considered circardian and infradian rhythmicity and the modulation of biological rhythms by pathophysiological disease-associated mechanisms. The development of respective approaches is of particular interest considering the detrimental impact of DBS on sleep patterns and quality, and the frequent link between ictogenesis and selected sleep or awakening phases in many patients. Thus, an individualized approach which takes biological rhythms into account can on one hand limit adverse effects of DBS and on the other hand better prevent or stop breakthrough seizures by adjusting stimulation to the situation and vigilance states. The adjusted stimulation algorithm applied in the case study comprised three levels with increasing stimulation intensity: (1) circadian basal stimulation during awakefulness and active phases with a day- and a night-time mode (13 Hz, 0.5 or 0.7 mA, respectively), (2) elevated stimulation during the patient's more seizure-prone sleep phases to protect from sleep-associated breakthrough seizures, controlled by activity/inactivity-assessing accelerometry (13 Hz, 1.3 mA), and (3) high-amplitude, high-frequency stimulation aiming to terminate seizures activity in case of breakthrough seizures (burst of 130 Hz, 1.5 mA) (64). The latter mode can be activated by the carer by a tap on the device on the forehead (detected via accelerometry) or by a tablet computer. Implantation and application of the described stimulation algorithm in the canine patient successfully prevented status epilepticus and reduced coherent cluster seizures during the follow-up phase of 7 months (64). Another closed-loop investigational device, sensing and stimulating both hippocampi and anterior nuclei of the thalamus, was implanted in two dogs with idiopathic epilepsy (120, 121). The authors reported that the device tracked successfully seizure activity, but did not report about how successful the device was in suppressing epileptic seizures. Lessons learned from these case studies in canines have now informed human trials. However, further randomized trials are also needed in veterinary medicine to explore if DBS should be developed as a clinical therapeutic tool despites its significant costs and the need of advanced neurosurgical expertise [a summary of the equipment needed and surgical approach can be found in the Supplementary Material of (64)]. In summary, these case studies provided proof-of-concept for adaptive devices combining physiological sensing of activity and vigilance states with a chronotherapy approach. The findings suggest that it is worthwhile to further explore the therapeutic potential and tolerability of multi-scale rhythmic brain stimulation approaches and highlights the dog's role as a translational model.

## Repetitive Transcranial Magnetic Stimulation

Transcranial magnetic stimulation (TMS) uses alternating magnetic fields to create a secondary electric field allowing for a non-invasive brain stimulation. Over the past decades repetitive TMS (rTMS) have increased clinical use with low frequency stimulation (<1 Hz) to induce reduced excitability or high frequency stimulation (>1 Hz) to achieve increased excitability (122). The principle behind this is mostly attributed to changes in synaptic plasticity in the form of long-term potentiation or depression (123). In epilepsy, rTMS focuses either on a precise epileptogenic zone or diffuse epileptogenic networks (124, 125). Cortical areas are mainly affected by the rTMS as its effect declines with the square of the distance from the coil; this is in contrast to other neurostimulation techniques such as DBS which can directly affect subcortical areas. Hence, epileptogenic networks located deeper than the cortex (e.g., cerebral or in particular thalamic nuclei) are less likely to be stimulated, unless the coil output is strong and/or the tissues between the coil and the brain (i.e., skull, muscles) are thin enough to allow penetration of the focused magnetic field up to these areas (126). However, studies have shown that rTMS can also have an impact on these subcortical areas through altering the function and connectivity of various neural networks (127–129).

Low frequency rTMS targeting a predetermined cortical area has been considered as a supportive therapy for suppression of seizures in refractory status epilepticus unresponsive to the conventional treatment options (130). Ictal rTMS in human patients provided promising results to abort ongoing prolonged seizures (ranging from few to 40–50 seizures per day) of human patients in inpatient or intensive care units. In a case report (131), one patient was treated with rTMS for 8 days (0.5 Hz, 60 min), which resulted in a marked clinical improvement successfully allowing the patient to be weaned off the respirator and sent to a rehabilitation clinic after discharge. In another study (132) similar improvement was achieved with only a single train of stimulation in one of the two patients (1 Hz, 20 min), whilst another patient (1 Hz, 30 min) responded with increased seizure frequency at 72 h post rTMS after a temporary improvement at 48 h. In another patient rTMS resulted in seizure freedom on lower doses anti-seizure medications after 11 days of stimulation (1 Hz, 10 min) (133). It should be noted that the improvements reported show quite heterogeneous periods ranging from hours to months.

Interictal rTMS, on the other hand, is applied at predetermined intervals and in structured sessions. The first pivotal study of interictal rTMS reported a transient improvement of about 38% reduced incidence of seizures per week in 9 patients during the 4 weeks post-treatment (0.33 Hz, 500 pulses of 2 trains per day, 5 consecutive days) (134). A later study reported improvements only in patients with single epileptic focus (2/4 patients) after a treatment that spanned 4 weeks (0.5 Hz, 100 pulses, applied biweekly), but not in patients with multiple foci (135). Whilst such a beneficial effect was not possible to be reproduced in another study with either single or multiple epileptic foci (136), the heterogeneous results were attributed to the differences in coil type, coil positioning, number and location of the epileptic foci (137, 138). A relatively recent controlled clinical trial (139) reported absence of any improvement after rTMS (0.5 Hz, 1,500 pulses/day, 10 weekdays) in patients with well-defined focal epilepsy during 10 weeks of follow-up period, regardless of the coil type used (8-shaped, circular or sham).

Differences in stimulation frequency (ranging 0.3 to 1 Hz), coil type (8-shaped, cone-shaped or round coils), output (>70% vs. <70%) and positioning (over epileptic focus, vertex or cerebellum) as well as stimulation period (days to weeks) and pattern (consecutive days or intermittent) in addition to the patient heterogeneity and small cohort sizes in clinical studies altogether hinder a direct systematic comparison and deduction of a standardized treatment protocol.

The first report on the use of rTMS in dogs with epilepsy was presented as an abstract during the 60th Annual Meeting of the American-Epilepsy-Society in 2006 (140). Although this study was a non-randomized uncontrolled trial and included only a very small number of subjects (*n* = 3), its preliminary results showed an increased seizure interval after stimulation compared to the baseline; however, further details on the outcome were not reported. Recently, a single-blinded randomized sham-controlled clinical trial was published by Charalambous et al. (65), which involved 12 dogs with drug-resistant idiopathic epilepsy. A round coil was used over the vertex to globally stimulate the cortex (1 Hz, 90 pulses, 18 trains/day, 5 consecutive days). Significant reductions in the monthly seizure frequency and monthly seizure day frequency were observed in the actively stimulated patients (7/12), but not in the sham treated patients (5/12). In a second trial, the sham group received active stimulation using the same parameters, which also resulted in a significant improvement. The positive effects lasted for 4 months, and no treatment-related side effects were reported. These results are quite encouraging compared to the discrepant reports in human studies. Due to practical reasons, canine patients, unlike human patients, invariably require sedation, which attenuates the extent of cortical excitation achieved by TMS (141). Although anesthetic drugs can suppress the neuronal activity (142–144), neuronal effects of rTMS have been shown in anesthetized rats (145). In an experimental study in dogs, an increase in the cerebral blood flow at the stimulation site was detected under both anesthesia and sedation, with higher but shorter increases in dogs under sedation (146). The study showed that, despite the effect of anesthesia and sedation on the neural networks, comparable and clinically relevant increases on the cerebral blood flow can be achieved in dogs when stimulated with rTMS.

## Seizure Detection and Forecasting and Its Application in Neurostimulation

A fundamental gap in epileptology is the lack of accurate seizure diaries. In fact, all pharmacologic and neurostimulation device studies to date have relied on patient diaries despite their unreliability (147, 148). While NeuroPace RNS and Medtronic Percept have recording capabilities, they do not reliably provide accurate seizure diaries (149, 150).

Recent device advances including continuous intracranial electroencephalography (iEEG) streaming, embedded and off-the-body detection algorithms and increasing on device data storage are poised to overcome this important engineering gap (151–153).

The potential importance of seizure forecasting is widely recognized (154). Evidence from RNS Neuropace Inc. investigations support that seizures are difficult to stop once they are detected on clinical iEEG macroelectrodes. In clinical practice this generally leads to using a highly sensitive detector resulting in >100 responsive electrical stimulations a day for optimal efficacy. Forecasting seizures with relatively good sensitivity has been demonstrated in canines (147, 155) and humans (148, 156) using continuously recorded iEEG. This has opened a potential new therapeutic window where neurostimulation or pharmacological treatments could be adjusted according to the probability of seizure occurrence (157).

The advances in device technology have yielded important insights into the generation of seizures. In particular, it is now well-established that seizures and seizure risk show multidien rhythms (158) in humans (159, 160) and canines (161). This important observation, that was first reported nearly 100 years ago (162), should prove useful for seizure forecasting and intelligent chronotherapy (64, 120).

## Future Perspectives

**Figure 2:**
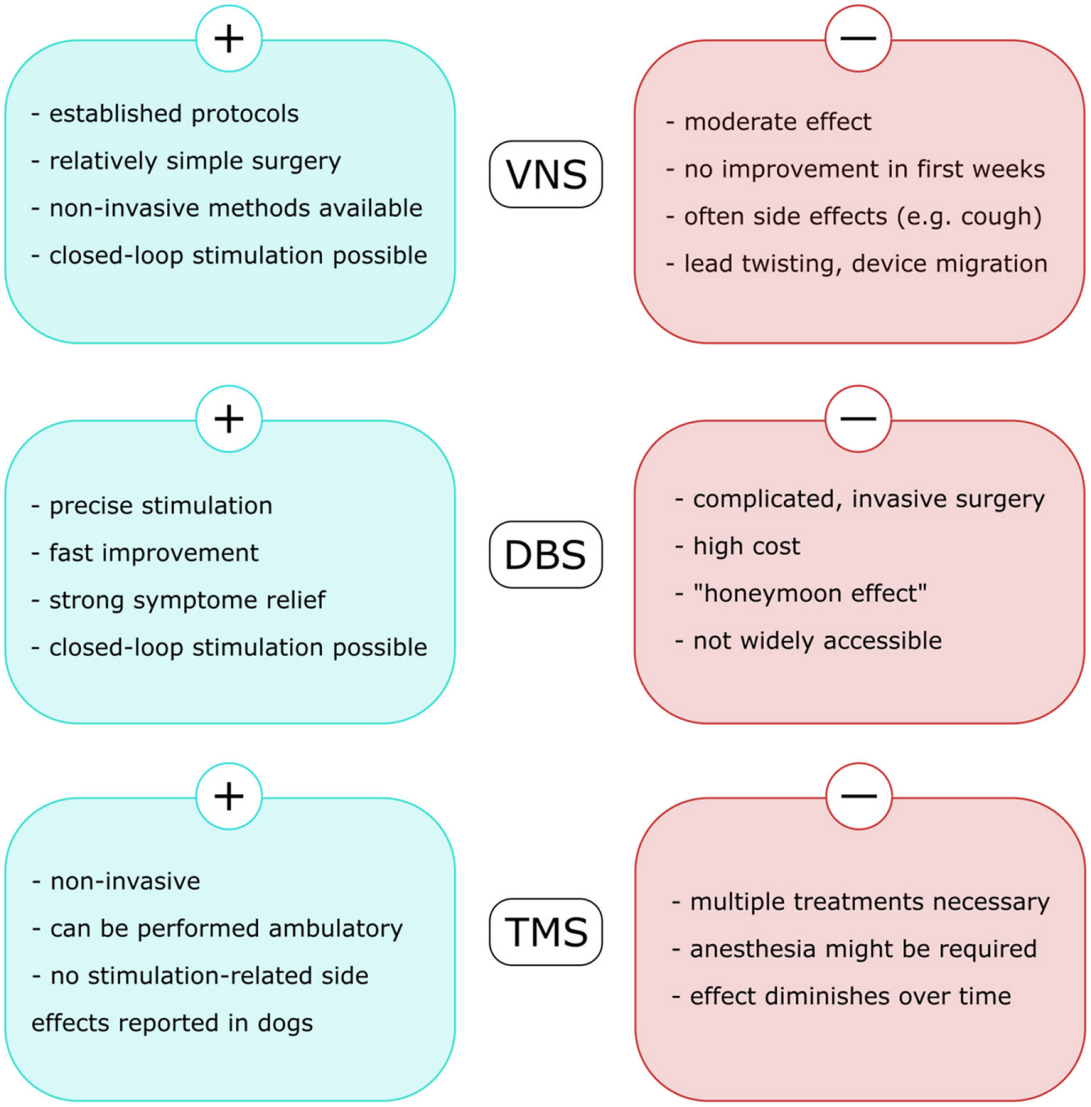
Advantages and challenges related to each of the neurostimulation methods used in veterinary medicine to treat drug-resistant epilepsy in dogs. VNS, vagus nerve stimulation; DBS, deep brain stimulation; TMS, transcranial magnetic stimulation.

**Figure 3:**
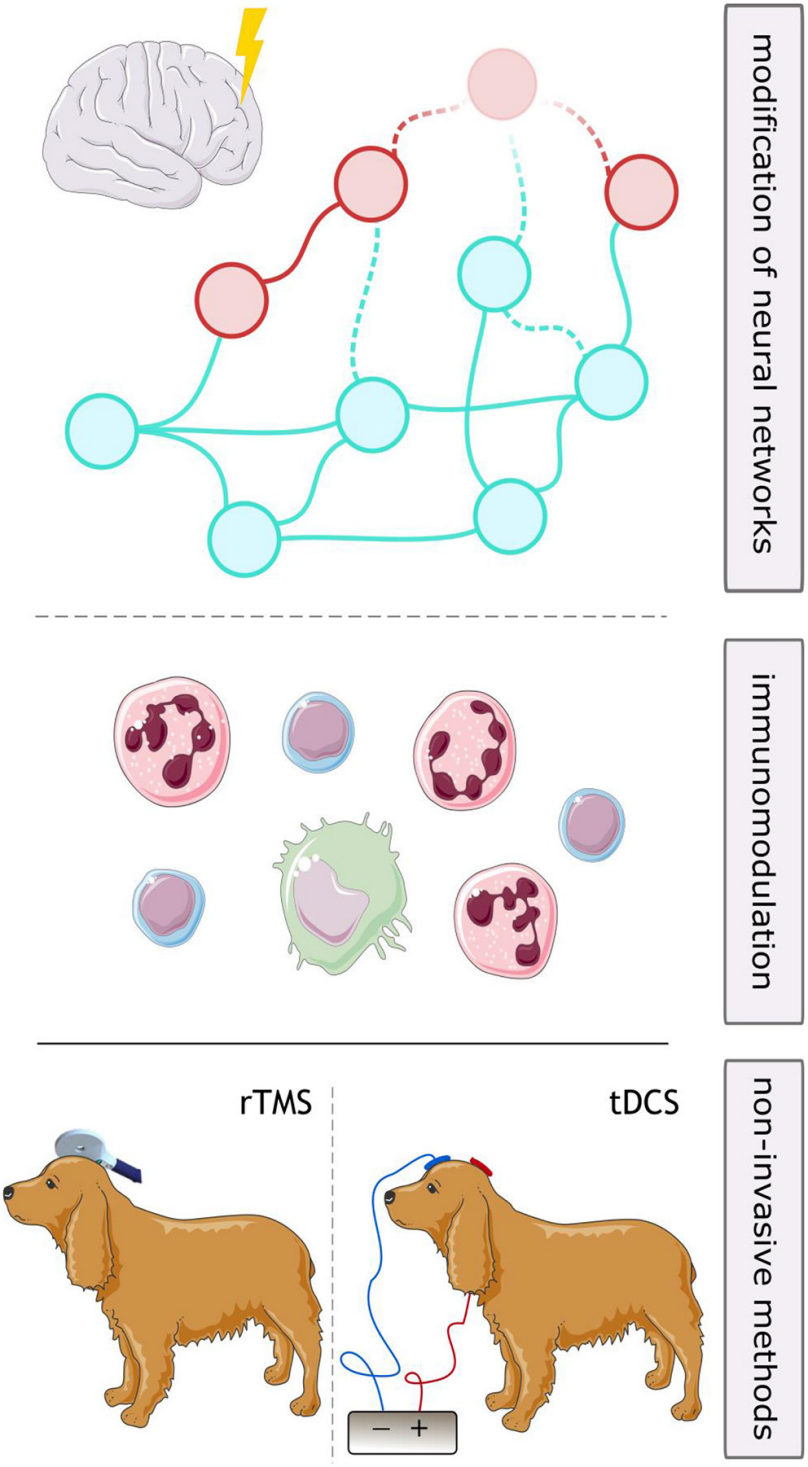
Future perspectives for neurostimulation in drug-resistant epilepsy in dogs. Long-term stimulation might lead to disease modifying effects through alterations in neuronal networks (upper part) or anti-inflammatory effects (middle part), which might be utilized to curb epileptogenic processes. Use of repetitive transcranial magnetic stimulation (rTMS) or transcranial direct current stimulation (tDCS) might both be considered non-invasive strategies for long-term stimulation in patients not eligible for surgery.

Neurostimulation (VNS, DBS, and RNS) are established therapies in human DRE. Transcutaneous VNS and TMS appear well-tolerated, but there are currently insufficient data to support the efficacy of any of these modalities for drug-resistant epilepsy (163). Although each of the described approaches possesses its specific advantages and challenges (Figure 2), they all proved to reduce seizure frequency and disease burden in both human and veterinary medicine. These methods are mostly associated with mild, often local side effects, therefore should be considered as alternative long-term treatment option of DRE in canine patients. However, the application of brain stimulation is currently rather limited to halting seizures on their onset, either in an open-loop or in a closed-loop manner. A reasonable next step in the research of neurostimulation in epileptology would be exploration of its anti-epileptogenic potential and possibility of disease modification (Figure 3).

Empirical evidence of the influence of electrical stimulation on epileptogenic process is already available from animal models. DBS performed in irregular intervals during interictal phases slowed progression of kindling-induced epileptogenesis and decreased generalized seizure duration in rats (164). High frequency DBS applied during 3 months in a macaque model of mesial temporal lobe epilepsy decreased levels of mRNA of genes involved in focal-adhesion and extracellular matrix-receptor interaction pathway (165), known to be up-regulated in epileptogenesis (166). Low frequency stimulation improved cognitive functions and memory during epileptogenesis in a kindling rat model (167), suggesting its influence on vast neuronal networks. In case of confirmation of these processes taking place in a canine brain, this might be of future interest for dogs following epileptogenic insults such as traumatic brain injury or virus encephalitis.

It is difficult to pinpoint, which exact mechanisms are involved in long-term outcomes of neurostimulation in epilepsy. Hypothetically, they could arise due to modifications in epileptic networks, their anti-inflammatory effects or due to other, more elusive processes, such as involvement of gut microbiota or anti-oxidative processes.

The effect on neuronal networks can be explained in the context of prolonged stimulation. The number of applied treatments may exceed the number of actual seizures and occur predominantly in the interictal period. Long-term iEEG recordings in patients with focal epilepsy undergoing chronic responsive neurostimulation system (RNS) therapy revealed reorganization of their brain networks: connectivity was lower between epileptic foci than in brain regions outside the foci (34). This effect was more prominent in patients with a better outcome in seizure reduction, which may suggest that neurostimulation helps disrupt pathological epileptogenic networks. However, epileptic networks could later re-adapt to the stimulation pattern, which might be responsible for the emergence of a “honeymoon phase” after the stimulation—this effect has been observed in patients with Parkinson's disease treated with DBS (168, 169). Therefore, if this change is permanent or why in some patients an alternate epileptogenic network re-organizes does require further research. In this context it needs to be considered that the outcome is also influenced by the parameters of the stimulation: e.g., in patients with Parkinson's disease, DBS performed with low frequency signals promoted circuit synchronization, whereas high frequency DBS suppressed synchronous activity (35). Long-term VNS resulted in changes in neural networks as well. Chronic VNS performed in naïve rats led to long-lasting increases of doublecortin-positive cells in the hippocampus as well as their dendritic complexity and expression of brain-derived neurotrophic factor (170), all of which are hallmarks of neuroplasticity. The data from patients additionally supports the evidence that VNS modulates neuronal networks into a less epilepsy-prone state (108). Moreover, unlike DBS, VNS does not induce a “honeymoon phase”—on the contrary, its effect seems to improve with time, which could indicate a beneficial influence of this stimulation mode on epileptic networks.

Inflammation is a process inseparably connected to epilepsy. Seizures can provoke production of pro-inflammatory cytokines, prostaglandins and chemokines by glia and neurons, by which they recruit immune cells from peripheral blood and lead to brain inflammation (171). Inversely, activation on innate immunity receptors causes rapid changes in ionic fluxes in neurons, which results in hyperexcitability and leads to onset or progression of a seizure (172). Brain inflammation also modifies expression of genes involved in production of neurotransmitter receptors, in neurogenesis and cell death and survivability (171, 172). This leads to network reorganization and changes in neuronal excitability, which can result in precipitation of the epileptogenic process.

The vagus nerve, as a part of the autonomic nervous system, is heavily involved in modulation of immune response (173). Stimulation of both vagal efferents and afferents has shown anti-inflammatory effects, attributed to cholinergic signaling (174). Experimental data supports positive effect of VNS on neuroinflammation in various animal disease models (175–178). Importantly, chronic VNS decreased levels of pro-inflammatory cytokines in hippocampus of rats with spontaneous recurrent seizures (178). Moreover, in a traumatic brain injury (TBI) rat model, VNS significantly suppressed expression of nuclear factor-kappa B (176), which is critically important for both inflammation and epileptogenesis (171). These findings could prove vital for prevention of disease development after epileptogenic insults.

Anti-inflammatory effects of DBS have also been established in animal models of epilepsy. DBS of ANT reduced blood-brain barrier disruption and albumin extravasation (179) as well as inflammation and apoptosis in rats with chemically induced status epilepticus (179, 180). It might suggest positive influence of stimulation on anti-inflammatory state of the brain is more pronounced than local inflammation caused by electrode insertion.

There might be other processes influencing to lesser extent the onset and progression of epileptogenesis, which might be targeted by brain stimulation. Recently, considerable insight has been gained into the role gastrointestinal microbiota plays in epilepsy (181). Even though short VNS did not alter gut microbiota composition in mice (182), repeated TMS of prefrontal cortex influenced rectal function of human volunteers, supposedly also affecting their microbiota (183). Another important epileptogenic factor is oxidative stress, leading to mitochondrial dysfunction and ionic dysbalance in neurons (184). Anti-oxidative effects have been reported in TMS in humans (185) and described in ischemic myocardiac injury in dogs (186), so it is plausible to assume they might also play a role in epileptic brains.

Canine patients with epilepsy have been included in clinical research involving three brain stimulation methods: VNS, DBS, and TMS. One of non-invasive stimulation methods used as treatment in humans with epilepsy and not researched in dogs as to date is transcranial direct current stimulation (tDCS). It utilizes weak (1–2 mA), constant, unidirectional flow of electrical charge applied to the scalp via electrodes mounted on a skin using an electrolytic contact medium (e.g. conductive gel) (187). The current modulates membrane potentials, leading to alteration of neuronal excitability. The effect of the stimulation depends on the direction and intensity of the applied current—anodal (positive) tDCS generally leads to increase of cortical excitability, while cathodal (negative) tDCS results in inhibition (187). Several studies in humans showed promising results including suppression of epileptiform discharges and decrease of seizure frequency following tDCS treatment (32, 187). Among adverse effects, minor skin itching and irritation at the stimulation site were reported (187). Considering lack of invasiveness, positive stimulation results, relatively short stimulation sessions (usually 20 min a day) and lack of serious side effects described, tDCS poses an excellent opportunity for canines with epilepsy (Figure 3).

To introduce new methodology into veterinary medicine and further establish existing ones, more clinical research in canines is needed. This would allow development of reliable protocols to improve the anti-seizure effect and avoid undesirable side effects, so that the neurostimulation becomes more effective and more safe for the patients. Equally important is further elucidation of the mechanisms governing respective stimulation approaches. So far, thanks to the basic research on dogs, it was possible to identify parameters for VNS in dogs (58) and describe its effect on seizure threshold and monoamine concentration (60). Nevertheless, further research is vital to better understand methods applied to the patients and ascertain the best possible management of refractory epilepsy.

## Author Contributions

MN, HV, HP, GW, TD, and MÜ: outline of the review. MN, HV, HP, GW, MÜ, MC, SB, and SM: writing of the manuscript. TD and HV: supervision. All authors contributed to the article and approved the submitted version.

## Funding

MN and MÜ are financed from ZK 17 Zukunftskolleg provided by the Austrian Science Fund (FWF – Der Wissenschaftsfonds). GW has received funding from National Institutes of Health (U01-NS073557, R01-NS92882, and UH2/3-NS95495) and the Epilepsy Foundation Epilepsy Innovation Institute My Seizure Gauge. This open access publication was funded by the Deutsche Forschungsgemeinschaft (DFG, German Research Foundation) - 491094227 Open Access Publication Costs and the University of Veterinary Medicine Hannover, Foundation.

## Conflict of Interest

GW has rights to receive future royalties from the licensing of technology to Cadence Neuroscience Inc, and has received research support from Medtronic, LivaNova, and was previously on the scientific advisory board of NeuroPace Inc. HV served as paid consultant in the field of epilepsy for Boehringer Ingelheim, CEVA animal health, Nestle Purina and served as contract researcher for: Nestle Purina, Desitin Pharma and Boehringer Ingelheim. HP received funding for consulting, talks and research collaborations from Eisai, Zogenix, Elanco, Roche, Exeed Epidarex, Arvelle and MSD. The remaining authors declare that the research was conducted in the absence of any commercial or financial relationships that could be construed as a potential conflict of interest.

## Publisher's Note

All claims expressed in this article are solely those of the authors and do not necessarily represent those of their affiliated organizations, or those of the publisher, the editors and the reviewers. Any product that may be evaluated in this article, or claim that may be made by its manufacturer, is not guaranteed or endorsed by the publisher.
